# Umap and Bismap: quantifying genome and methylome mappability

**DOI:** 10.1093/nar/gky677

**Published:** 2018-08-30

**Authors:** Mehran Karimzadeh, Carl Ernst, Anshul Kundaje, Michael M Hoffman

**Affiliations:** 1 Princess Margaret Cancer Centre, M5G 1L7, Toronto, ON, Canada; 2 Department of Medical Biophysics, M5G 1L7, University of Toronto, Toronto, ON, Canada; 3 Vector Institute, M5G 1M1, Toronto, ON, Canada; 4 Department of Human Genetics, McGill University, H3A 0C7, Montreal, QC, Canada; 5 Department of Genetics, Stanford University, 94305-9025, Stanford, CA, USA; 6 Department of Computer Science, Stanford University, 94305-5120, Stanford, CA, USA; 7 Department of Computer Science, University of Toronto, M5S 2E4, Toronto, ON, Canada

## Abstract

Short-read sequencing enables assessment of genetic and biochemical traits of individual genomic regions, such as the location of genetic variation, protein binding and chemical modifications. Every region in a genome assembly has a property called ‘mappability’, which measures the extent to which it can be uniquely mapped by sequence reads. In regions of lower mappability, estimates of genomic and epigenomic characteristics from sequencing assays are less reliable. These regions have increased susceptibility to spurious mapping from reads from other regions of the genome with sequencing errors or unexpected genetic variation. Bisulfite sequencing approaches used to identify DNA methylation exacerbate these problems by introducing large numbers of reads that map to multiple regions. Both to correct assumptions of uniformity in downstream analysis and to identify regions where the analysis is less reliable, it is necessary to know the mappability of both ordinary and bisulfite-converted genomes. We introduce the Umap software for identifying uniquely mappable regions of any genome. Its Bismap extension identifies mappability of the bisulfite-converted genome. A Umap and Bismap track hub for human genome assemblies GRCh37/hg19 and GRCh38/hg38, and mouse assemblies GRCm37/mm9 and GRCm38/mm10 is available at https://bismap.hoffmanlab.org for use with genome browsers.

## INTRODUCTION

**Figure 1.**
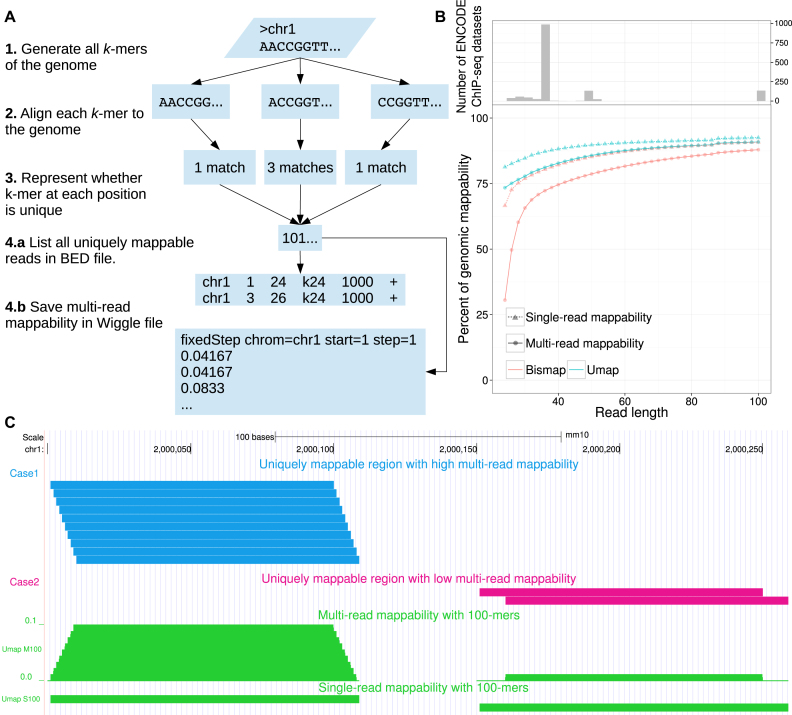
Mappability of the genome by Umap. (**A**) The Umap workflow identifies all unique *k*-mers of a genome given a read length of *k*. (**B**) Mappability of the human genome and methylome for read lengths between 24 and 100. (**C**) All of the uniquely mappable reads in two regions with high and low multi-read mappability are shown. In Case 1 (blue), all possible reads covering the region are uniquely mappable. In Case 2 (magenta), only two reads out of 10 are uniquely mappable.

High-throughput sequencing enables low-cost collection of high numbers of sequencing reads but these reads are often short. Short-read sequencing limits the fraction of the genome that we can unambiguously sequence by aligning the reads to the reference genome (Figure 1B). Still, we can identify much of the regulatory regions of the genome, such as transcription factor binding sites, histone modifications and other important regulatory regions. However, reads that are ambiguously mapped produce a false positive signal that misleads analysis. Some regions of the genome with low complexity including repeat elements are not uniquely mappable at a given read length. Other regions overlap few uniquely mappable reads, and consequently the mappability is low. To map the regions with low mappability, a high sequencing depth is required to assure that sequencing reads completely overlap with few uniquely mappable reads in that region. If sequencing depth is low and genomic variation or sequencing error is high, the signal from a low mappability region is biased by reads falsely mapped to that region.

Most short-read alignment algorithms determine if any read maps to one or more regions in the genome. However, one must consider this in context of the surrounding regions, even if a read maps uniquely. A single nucleotide change might change a read from uniquely mappable to not. A uniquely mappable read that aligns to a region with low mappability has a higher chance of not mapping uniquely due to genetic variation or sequencing error.

In bisulfite sequencing, this problem increases. Bisulfite treatment reduces unmethylated cytosine to uracil (sequenced as T), while 5-methylcytosine remains intact (sequenced as C). Bisulfite treatment significantly increases the number of repeated short sequences in the genome. Many regions uniquely mappable in an unmodified genome no longer uniquely map after bisulfite conversion. Incorrect mapping of bisulfite sequencing reads creates a false methylation signal that can bias downstream analysis and interpretation. When confounding factors such as read length, sequencing depth, or mutation rate differ among cases, this bias becomes even more evident.

In an unmodified human genome, 18.7% of the 24-mers do not map uniquely (Figure 1B). This quantity increases to 33.5% for a bisulfite-converted genome (Figure 1B). In certain cases, the difference between a uniquely mappable and a non-uniquely mappable read can be only 1 nt. Sequencer base-calling errors and genetic variation often affect alignment, but we cannot comprehensively account for them. These biases further exacerbate alignment when the read length is shorter, emphasizing the importance of considering genomic mappability in any analysis involving short-read sequencing. While previous tools such as the GEM-mappability software (1) identify mappability of the genome, no existing software solves the methylome mappability problem. In addition, existing tools prove difficult to use or lack available source code. To solve this problem, we developed the Umap software, with a bisulfite-mappability extension called Bismap.

## MATERIALS AND METHODS

### Single- and multi-read mappability

Umap identifies the uniquely mappable reads of any genome for a range of sequencing read lengths. The Bismap extension of Umap produces uniquely mappable reads of a bisulfite-converted genome. Both Umap and Bismap produce an integer vector for each chromosome that defines the mappability for any region and can be converted to a browser extensible data (BED) file. One way to assess mappability of a genomic region is by the ‘single-read mappability’—the fraction of that region that overlaps with at least one uniquely mappable *k*-mer. For a single base pair, the single-read mappability is 1 (the base pair overlaps at least one uniquely mappable *k*-mer) or 0 (the base pair does not). Regardless of whether the base pair lies at the beginning, middle or end of a uniquely mappable *k*-mer, any overlap is sufficient to make the single-read mappability 1.

Analysis of sequencing data involves inferences about a base’s genetic or regulatory state from observations of all reads overlapping that base. Therefore, we must consider the mappability of all reads overlapping a position or region, when estimating how many mapped reads we might expect. Single-read mappability assumes that uniquely mappable reads are uniformly distributed in the genome, while in reality we observe frequent localized enrichment of uniquely mappable reads.

A region can have 100% single-read mappability, but a below-average number of uniquely mappable reads that can overlap that region (Figure 1C). For example, a 1-kbp region with 100% single-read mappability can be mappable due to a minimum of 10 unique non-overlapping 100-mers or a maximum of 1000 − 1 + 100 = 1099 unique maximally overlapping 100-mers. Therefore, we define the ‘multi-read mappability’—the probability that a randomly selected *k*-mer in a given region is uniquely mappable. For the genomic region *G*_*i*:*j*_ starting at *i* and ending at *j*, there are *j* − *i* + *k* different *k*-mers that overlap with *G*_*i*:*j*_. The multi-read mappability of *G*_*i*:*j*_ is the fraction of those *k*-mers that are uniquely mappable (Figure 1C). Similarly, for any base pair in the genome, multi-read mappability is the number of unique *k*-mers overlapping that base pair divided by *k*.

### Mappability of the unmodified genome

Umap uses three steps to identify the mappability of a genome for a given read length *k* (Figure 1A). First, it generates all possible *k*-mers of the genome. Second, it maps these unique *k*-mers to the genome with Bowtie (2) version 1.1.0. Third, Umap marks the start position of each *k*-mer that aligns to only one region in the genome. Umap repeats these steps for a range of different *k*-mers and stores the data of each chromosome in a binary vector *X* with the same length as the chromosome’s sequence. For read length *k, X*_*i*_ = 1 means that the sequence starting at *X*_*i*_ and ending at *X*_*i* + *k*_ is uniquely mappable on the + strand. Since we align to both strands of the genome, the reverse complement of this same sequence starting at *X*_*i* + *k*_ in the − strand is also uniquely mappable. *X*_*i*_ = 0 means that the sequence starting at *X*_*i*_ and ending at *X*_*i* + *k*_ can be mapped to at least two different regions in the genome.

Eventually, Umap merges data of several read lengths to make a compact integer vector for each chromosome (Figure 1A, step 3). In this vector, non-zero values at position *X*_*i*_ indicate the smallest *k*-mer that positions *X*_*i*_ to *X*_*i* + *K*_ is uniquely mappable with, where *K* is the largest *k*-mer in the range. For example, *X*_*i*_ = 24 means that the region *X*_*i*_ to *X*_*i* + 24_ is uniquely mappable. This also means that any read longer than 24 nt that starts at *X*_*i*_ is also uniquely mappable.

Umap translates these integer vectors into six-column BED files for the whole genome (Figure 1A, step 4). Additionally, Umap can calculate single-read mappability and multi-read mappability for specified regions in any input BED file.

Although Bowtie can align with mismatches, here we do not use this capability. By defining mappability with exact matches only, we provide baseline identification of regions that are not uniquely mappable no matter how high the sequencing coverage is. Nonetheless, the Umap software allows users to change alignment options, including mismatch parameters.

### Mappability of the bisulfite-converted genome

**Figure 2.**
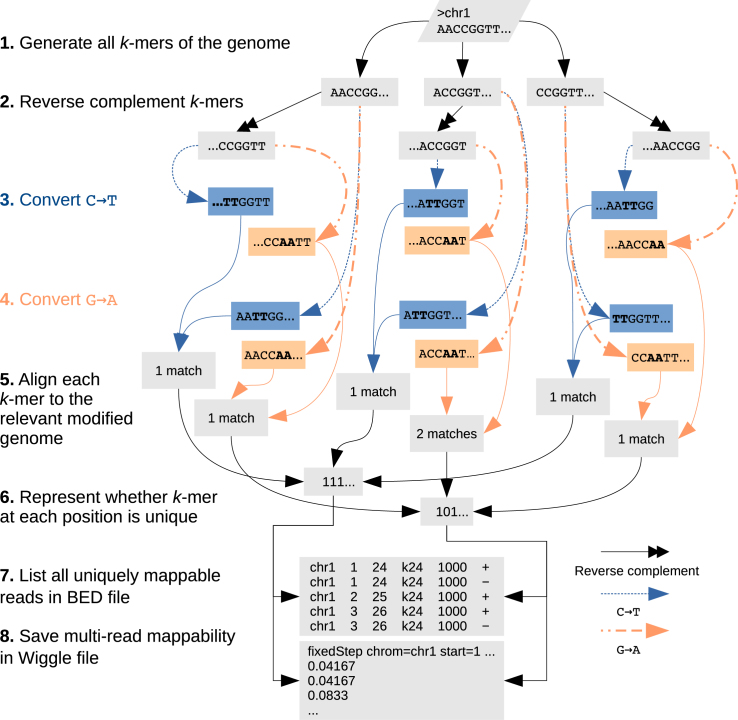
Mappability of the methylome by Bismap. Bismap identifies uniquely mappable *k*-mers of a bisulfite-converted genome. It simulates the same changes that may occur in bisulfite treatment on the + strand (C→T) and − strand (G→A). To account for sequence of the − strand, we generate an extra set of reverse-complemented chromosomes and then simulate bisulfite conversion on these chromosomes. We do not simulate reverse complementation after bisulfite conversion, because the experimental protocol does not involve post-conversion DNA amplification. We then align *k*-mers by disabling complement search and combine the resulting data to quantify the mappability of a bisulfite-converted genome.

To identify the single-read mappability of a bisulfite-converted genome, we create two altered genome sequences (Figure 2). In the first sequence, we convert all cytosines to thymine (C→T). In the other sequence, we convert all guanines to adenine (G→A). Our approach follows those of Bismark (3) and BWA-meth (https://arxiv.org/abs/1401.1129). We convert the genome sequence this way because bisulfite treatment converts unmethylated cytosine to uracil, which is read as thymine. Similarly the guanine that is base-pairing with the unmethylated cytosine in the − strand converts to adenine. These two conversions, however, never occur at the same time on the same read. We identify the uniquely mappable regions of these two genomes separately and then combine the data to represent the single-read mappability of the + and − strands in the bisulfite-converted genome. For an unmodified genome, however, the mappability of the + and − strand is identical by definition.

Bismap requires special handling of reverse complementation of C→T or G→A converted genomes. Conversion of C→T on the sequence 5′-AATT**CC**GG-3′ produces 5′- AATT**TT**GG -3′. In the Bowtie index, the reverse complement of the latter would be 5′- CCAAAATT-3′. For the purpose of identifying the mappability of the bisulfite-converted genome, however, we expect the reverse complement to be derived from the original converted sequence, yielding 5′- **CC**GGAATT-3′, and then after C→T conversion, 5′- **TT**GGAATT-3′. Both + and − strands undergo bisulfite treatment simultaneously, and there is no DNA replication to create new reverse complements after bisulfite treatment. To handle this issue, Bismap creates its own reverse complemented chromosomes and suppresses Bowtie’s usual reverse complement mapping.

Umap and Bismap each take ∼200 core-h on a 2.6 GHz Intel Xeon CPU E5-2650 v2 processor and <500 MB of memory to run for some read length in GRCh38. This is a massively parallelizable task, so on a computing cluster with 400 cores, the task takes only 30 min of wall-clock time.

### ENCODE ChIP-seq experiments

We downloaded ENCODE (4) chromatin immunoprecipitation-sequencing (ChIP-seq) FASTQ files from the ENCODE Data Coordination Center (https://www.encodeproject.org) and aligned them to GRCh38 using Bowtie 2 (5). We switched to Bowtie 2 for this analysis because it supports gapped alignment, which we did not need for mappability calculations.

We used Samtools (6) to remove duplicated sequences and those with a mapping quality of <10. This assures that the probability of correct mapping to the genome for any read is >0.9. Pooling replicates from the same experiment, we used MACS (7) version 2 with the options ‘- -nomodel - -q-value 0.001’ to identify ChIP-seq peaks. At last, Umap measured single-read mappability and multi-read mappability within the peaks.

### CpG islands

We downloaded CpG islands for GRCh38 from the University of California Santa Cruz (UCSC) Genome Browser (9) (http://hgdownload.soe.ucsc.edu/goldenPath/hg38/database/cpgIslandExt.txt.gz). We then annotated CpG features around the CpG islands following published definitions (8,10) (Table 1). Then, we used Umap and Bismap to measure mappability across these annotations.

**Table 1.** CpG annotations

Annotation	Definition
CpG island	As annotated by UCSC Genome Browser
CpG shore	2-kbp area surrounding CpG islands
CpG shelf	2-kbp area surrounding CpG shores
CpG resort	Collection of islands, shores and shelves

### Whole-genome bisulfite sequencing analysis

First, we obtained datasets of whole-genome bisulfite sequencing of murine mammary tissues (11) from the Sequence Read Archive (accession numbers: SRR1946823, SRR1946824, SRR1946819 and SRR1946820). Second, we trimmed Illumina TruSeq adapters from FASTQ files with Trim Galore (https://www.bioinformatics.babraham.ac.uk/projects/trim_galore). Third, for each experiment, we break down sequencing reads to produce two different FASTQ files with read lengths of 50 and 100 bp. For example, if the read length of an experiment is 182 bp and we want to generate a FASTQ file with read length of 50 bp, we would only use the first 50 bp and discard the rest. We aligned these modified FASTQ files with BWA-meth (https://arxiv.org/abs/1401.1129) to the GRCm38 genome. We removed duplicate reads or those with a mapping quality <10. We extracted CpG-context methylation using PileOMeth (https://github.com/dpryan79/MethylDackel). We use BSmooth (12) (version 0.4.2) for identifying differentially methylated regions. At last, we used Bismap to measure mappability of differentially methylated regions with at least four CpG dinucleotides.

### Other methylation assays

DiseaseMeth (13), a human methylation database, provides access to 17 024 methylation datasets from 88 different human diseases. These data are a collection of experiments using various platforms, including 2728 assays using the Illumina Infinium HumanMethylation27 (27K) BeadChip and 9795 assays using the Illumina Infinium HumanMethylation450 (450K) BeadChip. To identify which 50-bp probe sequences of the 27K (https://support.illumina.com/downloads/humanmethylation27_product_support_files.html) and 450K arrays (https://support.illumina.com/downloads/infinium_humanmethylation450_product_files.html) do not map uniquely to the GRCh37 genome, we measured single-read mappability with Umap. To identify which probes do not map uniquely after bisulfite conversion, we measured single-read and multi-read mappability with Bismap. In addition, we examined whether the exact 50-mer probe sequence mapped uniquely.

DiseaseMeth also contains 71 experimental datasets using reduced representation bisulfite sequencing (RRBS) (14). For CpG dinucleotides captured in RRBS experiments and annotated by DiseaseMeth, we examined the multi-read mappability for read lengths of 24, 36, 50 and 100 bp.

### Umap and Bismap track hub

We used read lengths of 24, 36, 50 and 100 bp to generate mappability tracks for unmodified and bisulfite-converted genomes of human (GRCh37 and GRCh38) and mouse (GRCm37 and GRCm38). For these genomes, we store single-read mappability (regions that overlap with *k*-mers that map uniquely) in bigBed format and per-base multi-read mappability in bigWig format as a track hub that can be loaded in the UCSC (9) or Ensembl (15) genome browsers. The UCSC Genome Browser GRCh38/hg38 mapping and sequencing track hub has Umap and Bismap tracks by default (https://genome.ucsc.edu/cgi-bin/hgTrackUi?db=hg38&g=mappability). The track hub contains one supertrack for Umap and one supertrack for Bismap. Umap software calculates single-read and multi-read mappability for any BED file as well. The track hub and software are available at https://bismap.hoffmanlab.org.

## RESULTS

### Mappability of ENCODE ChIP-seq peaks

**Figure 3.**
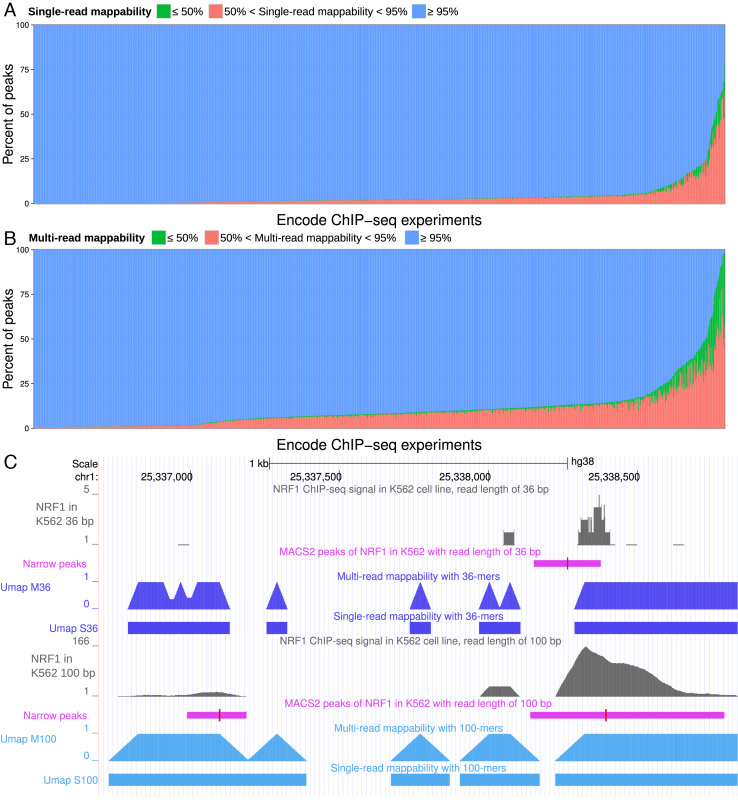
Mappability of ChIP-seq peaks in 1193 ENCODE datasets. (**A**) Single-read mappability and (**B**) multi-read mappability for narrow peaks identified in ENCODE ChIP-seq datasets. (**C**) An NRF1 narrow peak identified by MACS (purple) that is not uniquely mappable in the experiment with read length of 36 bp. The red bar in peaks indicates the summit. Signal tracks (gray) show two different replicates of this ChIP-seq experiment in K562 chronic myeloid leukemia cells (ENCODE accessions ENCSR000EHH and ENCSR494TDU, with read lengths of 36 and 100 bp, respectively). Umap tracks show single-read and multi-read mappability for two different read lengths of 36 and 100 bp.

ChIP-seq identifies proteins present in chromatin at particular loci and often involves short-read sequencing. The ENCODE Project (4) has performed around 1200 ChIP-seq assays on ∼200 chromatin-binding factors in more than 60 different human cell types. To show how mappability affects downstream analysis of experiments such as ChIP-seq, we quantified the mappability of narrow peaks identified in ENCODE ChIP-seq experiments. Among 1193 experiments, most peaks map uniquely. For some experiments, however, a high number of peaks overlap with non-uniquely mappable regions. Most of these experiments correspond to ChIP-seq of histone modifications with read lengths from 24 to 36 bp. There are two ENCODE NRF1 ChIP-seq experiments in K562 with 36 bp (ENCSR000EHH) and 100 bp (ENCSR494TDU and ENCSR998AJK) read lengths. For ENCSR000EHH among the 3994 peaks called by MACS2, 219 extend into a region that is not uniquely mappable. Although the ChIP-seq signal is completely within a uniquely mappable region, MACS2 identifies a much broader peak than is warranted (Figure 3C).

### Mappability of CpG islands

CpG islands substantially overlap transcription start sites and differentially methylated regions (8). Because CpG islands have a high number of CpGs, they are highly affected by bisulfite conversion. Thus, we investigated CpG islands and the neighboring CpG shores and CpG shelves.

Even with a relatively long read length of 100 bp, 3 059/167 694 CpG annotations have zero uniquely mappable bases, as calculated by Bismap. For shorter read lengths, even more of the bisulfite-converted genome lacks unique mapping. For a read length of 100 bp, 26 510 CpG annotations are not uniquely mappable with Bismap. This represents 15.8% of all CpG annotations. The average single-read mappability of CpG annotations that are not uniquely mappable is 68.8%.

CpG islands and regions around them are often not uniquely mappable, to a lesser extent, in an unmodified genome. For example, the average single-read mappability of 15 776 CpG annotations that are not uniquely mappable in the unmodified genome is 60% with a read length of 100 bp. This is substantially lower than the average single-read mappability of the genome (92%). Also, there are 631 CpG islands that have some overlap with uniquely mappable regions of the unmodified genome, but are not uniquely mappable in the bisulfite-converted genome.

**Figure 4.**
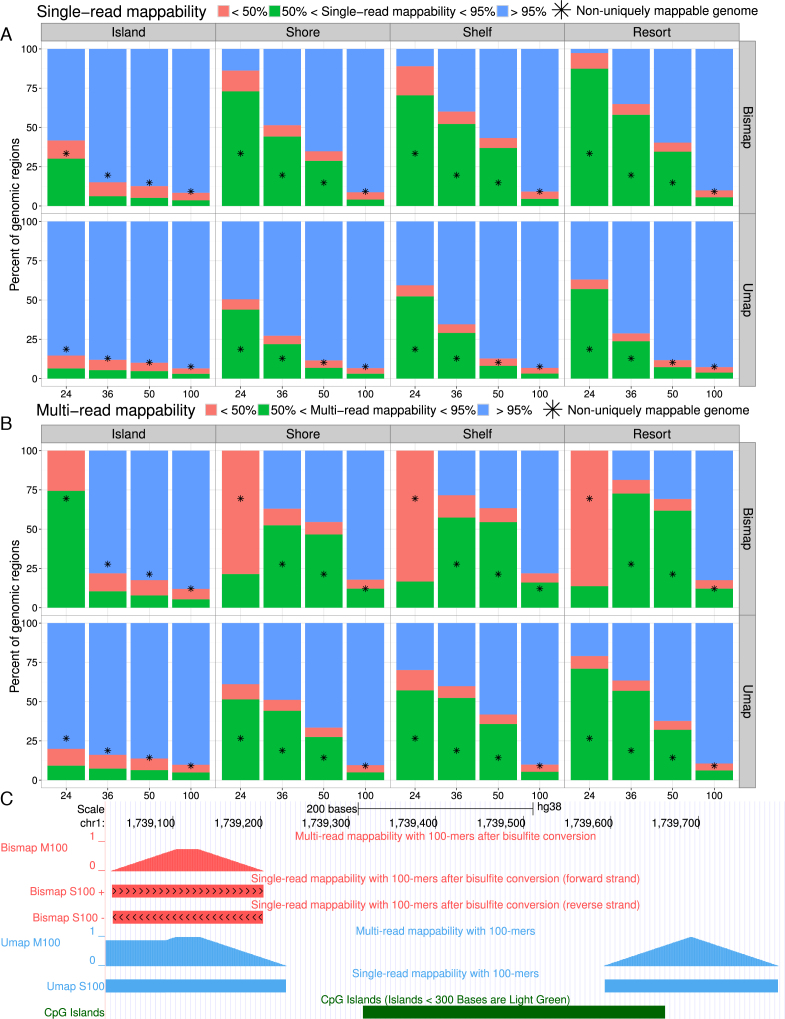
Mappability of the CpG island annotations. (**A**) Single-read mappability and (**B**) multi-read mappability of CpG islands, CpG shores, CpG shelves and CpG resorts for a variety of read lengths. For comparison, asterisks indicate the average mappability of the whole genome at each read length. (**C**) A CpG island that is not uniquely mappable with a read length of 100 bp by Umap and Bismap. In Bismap single-read mappability tracks, chevrons pointing right indicate mappability of the + strand and chevrons pointing left indicate mappability of − strand. Multi-read mappability is calculated based on reads that are uniquely mappable on both + strand and − strand.

The difference in genomic mappability and CpG island annotation mappability is even more extensive for shorter read lengths. For example, for a read length of 24 bp, more than 96.84% of CpG island annotations are not uniquely mappable, but the percent of the genome that is not uniquely mappable is only 30% (Figure 4).

### Mappability of differentially methylated regions

Many studies measure differences in methylation associated with a disease phenotype. These studies test whether each CpG’s methylation status correlates with the phenotype. Collective difference of CpG dinucleotides in a given region, however, may provide higher statistical power in assessing the association of methylation profile with disease states (16). Cluster of CpG dinucleotides are also a more predictive feature of disease states than differences in individual CpGs (16). BSmooth (12) is one of the tools that identifies differentially methylated regions by estimating a smoothed methylation profile.

**Figure 5.**
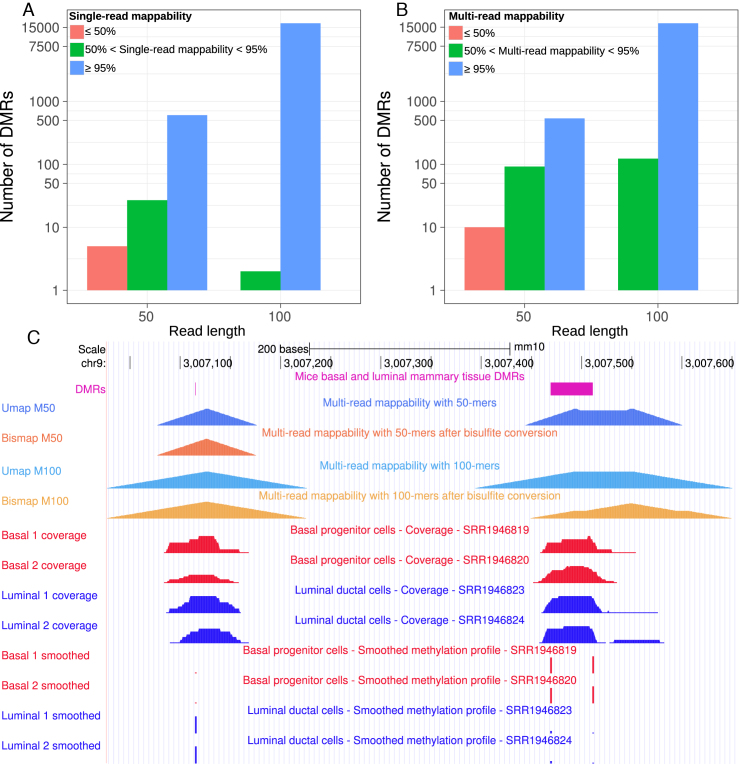
Mappability of differentially methylated regions of mice mammary basal and luminal alveolar tissues. (**A**) Single-read and (**B**) multi-read mappability of differentially methylated regions. (**C**) A differentially methylated region identified with 50-nt sequencing reads that are not uniquely mappable (purple). None of the sequencing reads that overlap this differentially methylated region uniquely map to the bisulfite-converted genome, although they all map uniquely to the unmodified genome.

We compared differences in CpG methylation of basal and luminal alveolar murine mammary tissues (11) using BSmooth (12). Out of a total of 383 119, CpG dinucleotides sequenced with a read length of 50 bp (see ‘Materials and Methods’ section), 306 of them are not uniquely mappable. For a read length of 100 bp, out of a total of 3 648 877 CpG dinucleotides, 411 are not uniquely mappable. For the same experimental setup, BSmooth identified 636 differentially methylated regions for a read length of 50 bp and 17 435 regions for a read length of 100 bp. For a read length of 100 bp, five differentially methylated regions were not uniquely mappable (single-read mappability <100%), while for a read length of 50 bp, 53 differentially methylated regions were not uniquely mappable (Figure 5). This is a proof of principle that differential methylation analysis can identify false signals that are not even uniquely mappable.

**Figure 6.**
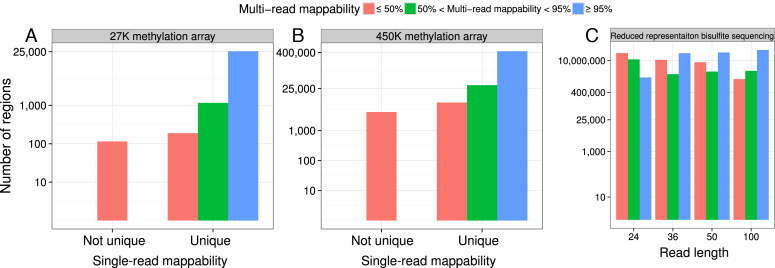
Mappability of targeted methylation assays. Multi-read mappability of probes in (**A**) the Illumina Infinium HumanMethylation27 (27K) BeadChip and (**B**) the Illumina Infinium HumanMethylation450 (450K) BeadChip. (**C**) Multi-read mappability of CpG dinucleotides found in DiseaseMeth RRBS datasets.

DiseaseMeth (13) catalogs publicly available methylome datasets, including 12 073 using array technologies. The cost-efficiency of these approaches has driven wide adoption. Many of these datasets, however, include probes with low mappability in the bisulfite-converted genome. The widely used Illumina Infinium methylation arrays use 50-bp probes capturing certain CpG dinucleotides. Out of the 27 578 probes in the Illumina Infinium HumanMethylation27 (27K) BeadChip, 377 do not map uniquely to GRCh37 and 115 do not map uniquely after bisulfite conversion. Additionally, 304 uniquely mappable probes have low multi-read mappability, meaning that single nucleotide polymorphisms or mutations can make the sequence unmappable or even result in probe multi-mapping (Figure 6A). Similarly, out of 485 512 probes in the Illumina Infinium HumanMethylation450 (450K) BeadChip, 84 are not uniquely mappable to GRCh37, 4146 are not uniquely mappable after bisulfite conversion, and another 12 744 uniquely mappable probes have low multi-read mappability (Figure 6B).

In addition, many publicly available RRBS datasets exist. In RRBS, only DNA fragments between 40  and 220 bp are selected. The majority of selected fragments, however, are ∼50 bp (17). Even with a read length of 100 bp, 408 384 (1.18%) of CpG dinucleotides in RRBS experiments of DiseaseMeth database did not map uniquely (Figure 6C).

### Limitations of paired-end sequencing

**Figure 7.**
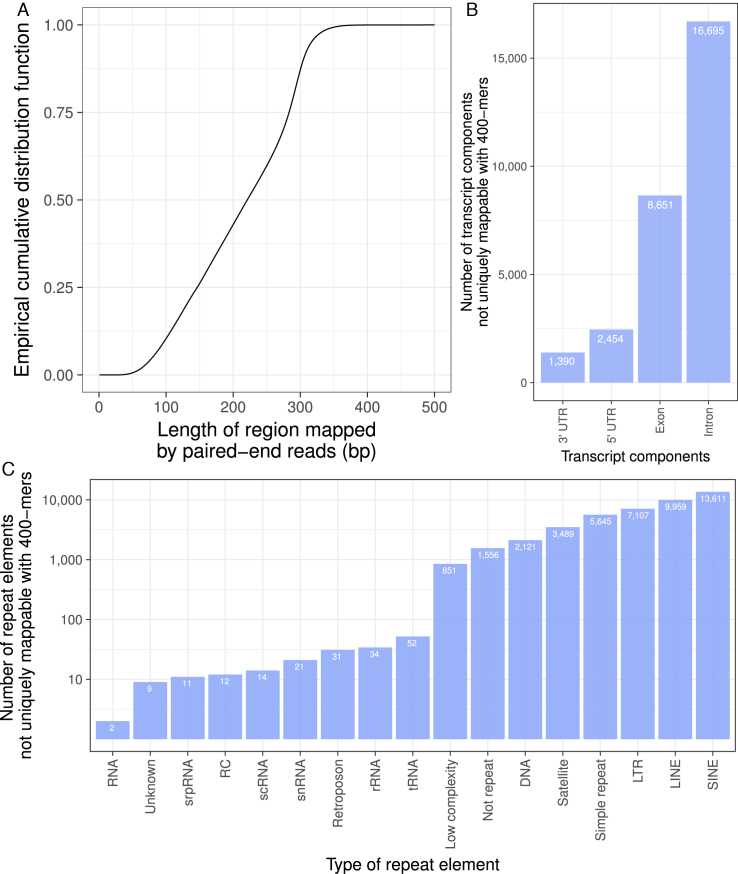
Limitations of paired-end sequencing. (**A**) Empirical cumulative distribution function of the length of a region mapped by paired-end reads in an experiment with 150 bp paired-end sequencing (ENCFF721VIZ). The plotted curve shows the proportion of regions (*y*-axis) that are shorter than some length (*x*-axis). This shows that 87.5% of mapped fragments are smaller than 300 bp. (**B**) Number of transcript components not uniquely mappable with 400-mers. (**C**) Number of RepeatMasker repeat elements not uniquely mappable with 400-mers. LTR, long terminal repeat; RC, rolling circle; rRNA, ribosomal RNA; scRNA, small conditional RNA; snRNA, small nuclear RNA; srpRNA, signal recognition particle RNA; tRNA, transfer RNA.

Paired-end sequencing links short reads to longer DNA fragments, increasing the frequency of unique mapping. To examine how paired-end sequencing might affect the need for mappability information, we examined an ENCODE whole genome bisulfite sequencing dataset with 150 bp paired-end reads (ENCFF721VIZ). Only 12.5% of sequenced fragments in that dataset were longer than 300 bp. Only 0.018% of fragments are longer than 400 bp (Figure 7A).

Because more than 99.9% of fragments in the paired-end sequencing dataset were 400 bp or shorter, we sought to identify genomic regions that cannot be uniquely mapped with 400-mers. A 400-bp fragment with reads from 150 bp ends may not provide all the information of a single 400-bp read. Here, we will assume that it does for a conservative estimate of which genomic regions map uniquely with paired-end reads.

We downloaded NCBI RefSeq gene annotations (18) using the UCSC Table Browser (19) (*Homo sapiens* annotation release 105, primary table: ncbiRefSeq, last updated: 29 November 2016). Out of 153 726 RefSeq gene annotations, 4521 overlap with genomic regions not uniquely mappable with 400-mers. Of these 4521 annotations, 3090 are not curated (XM and XR RefSeq IDs) while 1431 are manually curated (NM and NR RefSeq IDs). These regions overlapped thousands of annotated untranslated regions, introns and exons (Figure 7B). We downloaded human pseudogenes (GENCODE Release 27 GRCh38.p10, ftp://ftp.sanger.ac.uk/pub/gencode/Gencode_human/release_27/gencode.v27.2wayconspseudos.gtf.gz) and found that 210 of 9002 predicted pseudogenes do not map uniquely with 400 bp *k*-mers.

We downloaded the RepeatMasker (RepeatMasker Open-4.0, http://www.repeatmasker.org) annotation of repeat elements (primary table: rmsk, last updated: 10 January 2014) using the UCSC Table Browser. Only 48 260 of the 5 524 462 repeat elements did not map uniquely with 400 bp *k*-mers.

In the whole human genome, 44 525 regions did not map uniquely with 400 bp *k*-mers. Most of these regions (42 969) overlap RepeatMasker repeat elements. These include different types of repeat elements, mostly short- and long-interspersed nuclear elements (30.57% SINEs and 22.37% LINEs). Some of the non-unique regions overlapped non-messenger RNA (0.31% overlap either of transfer RNA, ribosomal RNA, small nuclear RNA, small conditional RNA or signal recognition particle RNA) or retrotransposons (0.06%, Figure 7C).

### Comparison of Umap and GEM-mappability

We compared Umap with the existing GEM-mappability software (1). GEM-mappability’s default parameters allow a 4% mismatch rate and require a minimum 80% match for a read to align. This means if a *k*-mer has pairwise alignment to a genomic region ≥96% but <100%, GEM maps it uniquely but Umap does not. Also, if a read maps to several genomic regions when allowing for 4% mismatch, GEM may report lower mappability of all those regions than Umap, if those regions map uniquely without mismatches. Each genomic position’s GEM-mappability score depends on how many times a *k*-mer starting at that position maps to the genome. GEM does not account for unique mapping that includes that position but starts upstream, unlike Umap’s single- and multi-read mappability scores. Therefore, we expect mappability scores for some genomic regions to differ between GEM and Umap.

**Figure 8.**
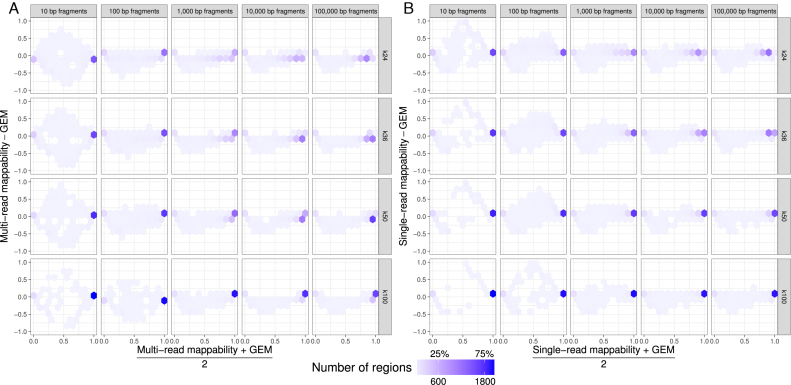
Bland–Altman density plots comparing Umap and GEM-mappability scores. For several read lengths (10, 100, 1000, 10 000 and 100 000) and *k*-mer sizes (24, 36, 50 and 100), we randomly selected 2400 regions (100 regions from each chromosome) and compared GEM-mappability with (**A**) Umap multi-read mappability and (**B**) Umap single-read mappability.

To compare GEM and Umap mappability scores, we repeatedly selected 2400 random regions (100 regions from each chromosome) of fixed lengths. We did this for five different region lengths (10, 100, 1000, 10 000 and 100 000), four different *k*-mer sizes (24, 36, 50 and 100), creating 5 × 4 = 20 comparison groups overall (Figure 8). Across all comparison groups, the minimum concordance correlation coefficient (20) between Umap multi-read mappability and GEM-mappability was 0.903 and the mean concordance correlation coefficient was 0.965. For single-read mappability, the minimum concordance correlation coefficient with GEM-mappability was 0.866 and the mean concordance correlation coefficient was 0.949.

Usually, the complete length of small regions mapped uniquely. For example, 86% of randomly selected 10-bp regions had both Umap single-read mappability and GEM-mappability scores of 1. In the remaining 10-bp regions, the Umap and GEM-mappability scores often disagreed. Umap single-read mappability was less than GEM-mappability in 5% of 10-bp regions, while Umap single-read mappability exceeded GEM-mappability in 1% of these regions.

For longer regions, the proportion of regions with the maximum score for either Umap or GEM dropped. Usually, Umap single-read mappability was higher than GEM-mappability, while Umap multi-read mappability was usually lower than GEM-mappability. In the regions with highest mappability, however, Umap multi-read mappability exceeds GEM-mappability. This happens because GEM-mappability only considers the uniqueness of a *k*-mer starting at a position, rather than also considering overlapping *k*-mers, like Umap.

## DISCUSSION

### The importance of considering mappability in analysis

In several examples, we showed how mappability must be considered in analysis of sequencing data. One needs to examine, however, the extent of genomic variation that affects mappability calculations. Genetic variants specific to each sample make it impossible to know the exact mappability. We introduced a measure called multi-read mappability for addressing this issue. Genomic regions with higher multi-read mappability are less prone to be biased by genetic variants and sequencing errors.

In ENCODE ChIP-seq experiments using short-read lengths, we found many examples where signal was within a uniquely mappable region but peaks identified by peak caller had substantial overlap with non-uniquely mappable regions. More than 50% of ChIP-seq data in the ENCODE Data Coordination Center use reads shorter than 36 bp. Consortia such as ENCODE and Roadmap have spent hundreds of millions of dollars to perform these experiments, which they will not repeat any time soon. This shows the importance of using the mappability information to analyze sequencing data, especially when the read length is short. In fact, we initially developed Umap as part of the ENCODE uniform analysis pipeline (4) to avoid such problems.

In Bismap, we convert all cytosines to thymines in the forward strand, and all guanines to adenines on reverse strand, just as alignment algorithms such as Bismark (3) or BWA-meth (https://arxiv.org/abs/1401.1129) do. In practice, chemical resistance or sample-specific genetic variation may retard bisulfite conversion. This makes it impossible to estimate the exact mappability for a bisulfite-converted sample. When performing bisulfite sequencing on different mouse strains, using the same reference genome for each introduces massive bias in bisulfite sequencing data analysis (https://doi.org/10.1101/076844). Ideally, one would align data from each strain to a reference genome specific to that strain. When one lacks a strain-specific reference genome, Bismap at least allows us to quantify how and where genetic variation affects reliability of bisulfite sequencing results. While Bismap assumes complete bisulfite conversion, Umap assumes none. By comparing the results of the two methods, we can understand the range of bisulfite-conversion effects on mappability.

While paired-end sequencing with lengths >100 bp has become more common, most publicly available datasets such as ENCODE have used shorter reads. Out of 3483 ENCODE ChIP-seq experiments, 3033 use single-ended sequencing, and 2228 have read lengths of 36 bp or shorter. Out of the 142 ENCODE RRBS datasets, 140 (98.6%) have a read length of 36 bp or shorter. In addition, commonly used array technologies such as the 450K array uses 50-bp probes and multi-read mappability of some of the probes is low. This allows multi-mapping due to genetic variation and decreases data quality in these regions as it has been noted before (21). Although only a small fraction of all probes do not map uniquely (1.8% in the 27K array and 0.87% in the 450K array), one must still use caution when interpreting methylation signal—or the lack thereof—in these regions. In fact, multi-mapping probes have lead to false discovery of autosomal sex-associated DNA methylation in at least one study (22).

In our analysis of whole genome bisulfite sequencing data of mouse mammary tissue, 0.08% of CpG dinucleotides were not uniquely mappable with 50-bp reads. We removed reads with a mapping quality <10 and only counted CpG dinucleotides that had a minimum coverage of three reads in all of the four different whole genome bisulfite sequencing datasets. Given this stringent filtering, the chance of observing any non-uniquely mappable read is 10^−12^, which is much less than our observation (0.1%). Most sequencing reads corresponding to these CpGs map uniquely to the unmodified genome, but not the bisulfite-converted genome. Such CpG dinucleotides must be excluded from analysis. RRBS usually involves filtering fragments to only include those that are 40 –220 bp, and most RRBS reads are 50 bp or less (17). This causes a major issue for mapping of these reads.

In paired-end sequencing, short regions from both ends of a longer fragment are sequenced. This provides a long read more likely to map uniquely to the genome. The length of these fragments varies considerably in size. One can still use Umap or Bismap to identify the mappability for a range of *k*-mers that represent the variation in fragment length of any given sequencing library. In a typical whole genome bisulfite sequencing assay with 150 bp paired-end reads, 87.5% of mapped reads cover <300 bp of the genome (Figure 7A). Even with a read length of 400 bp, *k*-mers overlapping 4521 of 153 276 RefSeq gene annotations (18) do not map uniquely.

In RNA-seq, gap alignment algorithms account for splicing. Different software and user defined parameters handle multi-mapping reads differently that can be a source of error. Some software does not remove multi-mapping reads. For example, Mortazavi *et al*. (23) assign multi-mapping reads in proportion to coverage of transcripts according to uniquely mappable reads, and Robert and Watson (24) recommend assigning multi-mapped reads to a group of genes instead of removing them. Other methods distribute reads among transcripts by modeling strand, mapped position, distribution of insert sizes (25) and transcript length (26) or pseudo-alignment of *k*-mers in a read to transcripts (27). Patro *et al*. (28) also account for sample-specific G+C-content and positional bias. These approaches accurately recover a significant portion of the data. In addition to using the above-mentioned features of sequencing reads, modeling local mappability of all possible matches to a sequencing read may improve accuracy of the current methods in transcript quantification.

### Other methods for mappability

GEM-mappability (1) quantifies the number of times each *k*-mer is mapped to the genome by allowing for mismatches (default 4%). If a *k*-mer is not uniquely mappable, but overlaps with other uniquely mappable *k*-mers, sequencing reads can map to that region and provide useful information about that genomic position. In addition to representing base-level mappability information, Umap single-read and multi-read mappability scores rely on unique mappability of downstream and upstream *k*-mers as well. Nevertheless, the mean concordance correlation coefficient (20) between GEM and Umap mappability is 0.949 (single-read) or 0.965 (multi-read) (Figure 8).

Bias Elimination Algorithm for Deep Sequencing (BEADS) (29) also defines a mappability measure that is obtained by identifying uniquely mappable 35-mers of the genome. Based on the assumption that each read identifies a longer 200-mer, BEADS extends uniquely mappable 35-mers to 200 bp and calculates the fraction of reads that span a given genomic position. BEADS uses a cut-off of 25% mappability to filter signals that might bias a study. Extending the 35-mer mappability to 200 bp, however, defines the exact mappability for neither 35-mers nor 200-mers.

PeakSeq (30) uses an algorithm similar to Umap and identifies the single-read mappability in 1-kbp windows of the genome. PeakSeq filters out ChIP-seq signals with low mappability in each window by comparing it to a simulated background of reads with Poisson distribution.

Model-based one and two Sample Analysis and inference for ChIP-Seq Data (MOSAiCS) (31) uses a mappability measure similar to multi-read mappability for preprocessing of data. While Umap’s multi-read mappability calculates the percent of uniquely mappable *k*-mers that span each nucleotide, MOSAiCS calculates the percent of extended uniquely mappable *k-*mers for calculating its mappability score. In comparison to other mappability measures, Umap’s multi-read mappability has the advantages of specificity to an exact read length and efficient calculation for any read length.

## DATA AVAILABILITY

The UCSC Genome Browser GRCh38/hg38 mapping and sequencing track hub has Umap and Bismap tracks by default (https://genome.ucsc.edu/cgi-bin/hgTrackUi?db=hg38&g=mappability). We have deposited in Zenodo the current version of our software (https://doi.org/10.5281/zenodo.800648) and the mappability data used in this project (https://doi.org/10.5281/zenodo.800645). In addition, the software (https://bitbucket.org/hoffmanlab/umap) is freely available under the GNU General Public License, version 3 (GPLv3).
